# The chronic effects of fish oil with exercise on postprandial lipaemia and chylomicron homeostasis in insulin resistant viscerally obese men

**DOI:** 10.1186/1743-7075-9-9

**Published:** 2012-02-07

**Authors:** Karin M Slivkoff-Clark, Anthony P James, John CL Mamo

**Affiliations:** 1 School of Public Health, Curtin Health Innovation Research Institute and the Australian Technology Network, Centre for Metabolic Fitness, Curtin University, Bentley Campus, Kent St, Perth 6102, Australia

## Abstract

**Background:**

Visceral obesity and insulin resistance are associated with a postprandial accumulation of atherogenic chylomicron remnants that is difficult to modulate with lipid-lowering therapies. Dietary fish oil and exercise are cardioprotective interventions that can significantly modify the metabolism of TAG-rich lipoproteins. In this study, we investigated whether chronic exercise and fish oil act in combination to affect chylomicron metabolism in obese men with moderate insulin resistance.

**Methods:**

The single blind study tested the effect of fish oil, exercise and the combined treatments on fasting and postprandial chylomicron metabolism. Twenty nine men with metabolic syndrome were randomly assigned to take fish oil or placebo for four weeks, before undertaking an additional 12 week walking program. At baseline and at the end of each treatment, subjects were tested for concentrations of fasting apo B48, plasma lipids and insulin. Postprandial apo B48 and TAG kinetics were also determined following ingestion of a fat enriched meal.

**Results:**

Combining fish oil and exercise resulted in a significant reduction in the fasting apo B48 concentration, concomitant with attenuation of fasting TAG concentrations and the postprandial TAG_IAUC _response (p < 0.05). Fish oil by itself reduced the postprandial TAG response (p < 0.05) but not postprandial apo B48 kinetics. Individual treatments of fish oil and exercise did not correspond with improvements in fasting plasma TAG and apo B48.

**Conclusion:**

Fish oil was shown to independently improve plasma TAG homeostasis but did not resolve hyper-chylomicronaemia. Instead, combining fish oil with chronic exercise reduced the plasma concentration of pro-atherogenic chylomicron remnants; in addition it reduced the fasting and postprandial TAG response in viscerally obese insulin resistant subjects.

## Background

The problem of obesity has become high priority globally. Nearly two thirds of deaths and 46% of the worldwide burden of disease are due to non-communicable, obesity-associated diseases [1]. In 2008 more than one third of the world's adults were overweight, which is a doubling of the prevalence since 1980 [2]. The phenotype of visceral obesity is associated with a cluster of related conditions called metabolic syndrome (MetS), which encompasses both lipid and non-lipid related disorders. These disorders include insulin resistance, pro-inflammatory and pro-thrombotic states and the emergence of an atherogenic lipoprotein profile. Amongst the latter, changes to postprandial lipoprotein kinetics and an increased fasting chylomicron remnant concentration are commonly reported and may contribute to increased coronary artery disease risk in viscerally obese subjects [3,4].

In MetS, higher rates of chylomicron production coupled with reduced clearance by high affinity pathways (i.e. the LDL receptor) can contribute to an accumulation of pro-atherogenic chylomicron remnants in plasma [5-9]. Moreover, several studies from our laboratory suggest that restoration of chylomicron homeostasis in insulin resistant obese subjects is more difficult to achieve than for hepatically derived lipoproteins. We found that an acute insulin infusion for six hours postprandially substantially lowered the apo B100 area under the curve but not the chylomicron apo B48 response [10]. In another study, chronically enhancing insulin sensitivity with the provision of metformin, or a peroxisome proliferator receptor antagonist (rosiglitazone) had no effect on fasting apo B48, or postprandial hyperchylomicronaemia [11]. Likewise, a 10 kg weight reduction following moderate energy restriction did not improve chylomicron homeostasis in a similar cohort [12]. Similarly, in dyslipidaemic diabetic subjects, others have found that the fasting apo B48 concentration was not significantly lowered by fluvastatin alone, but when fish oil was concurrently administered, the change was significant [13]. Based on these findings, we suggest that interventions designed to regulate lipoprotein kinetics may need to be more aggressive to modulate the chylomicron fraction, compared to interventions that reduce hepatically derived apo B.

There is interest in unsaturated fatty acids (FA), particularly the n-3 FA from fish oil in lipoprotein metabolism since they suppress secretion of apo B100 by the liver and promote plasma clearance via high affinity pathways [14-16]. Evidence from animal experiments shows that the chylomicron secretion rate may be up-regulated by habituating to a higher fat diet [17], and the type of fat can also influence chylomicron secretion [18] or fasting concentrations [19]. The n-3 FA could result in secretion of fewer chylomicrons because they significantly suppress intestinal TAG production in several experimental models of the intestine [20,21]. Furthermore receptor-mediated uptake of chylomicron remnants is significantly enhanced if the particles are enriched in n-3 FA [22]. However, how these n-3 related mechanisms translate into changes in human chylomicron homeostasis is yet to be resolved [23].

Human studies by Gill and colleagues [24] also suggest that postprandial lipoprotein metabolism is improved by exercise in otherwise healthy subjects. They reported in middle-aged men that a single bout of treadmill walking substantially enhanced apo B100 metabolism of the VLDL_1 _fraction concomitant with a slight reduction in postprandial apo B48 concentration. It is reasonable to suggest that a more sustained exercise regimen will have a greater effect on apo B48 metabolism due to progressive improvements in muscle vascularisation and greater conversion of TAG-rich lipoproteins to the high- uptake remnant form [25]. Improvements in insulin sensitivity associated with exercise may also stimulate high affinity remnant clearance pathways that are sufficient to promote chylomicron remnant clearance. However, the effects of a chronic exercise regimen, specifically on postprandial apo B48 metabolism in insulin resistant subjects are not reported.

Therefore we intended to investigate the combined interventions of fish oil and exercise on chylomicron metabolism in viscerally obese, insulin resistant men using a chronic study design. The protocol aimed to achieve an exercise volume consistent with optimising metabolic effects [26], and participation rates. The second part of our hypothesis is based on evidence that the n-3 FA have potential to modulate lipoprotein kinetics, at the level of clearance and possibly also secretion. Furthermore acute exercise may also alter apo B48 concentrations [24], but the long-term effect of exercise combined with fish oil, on chylomicron metabolism has not been previously explored in these subjects.

## Methods

### Participants and protocol

Sedentary overweight or obese men aged 32 to 65 years were recruited based on having MetS, a low physical activity level and absence of dietary oil supplementation or fish consumption. MetS was classified as having three or more risk factors specified by the ATP III guidelines [27]. Subjects had a BMI > 27 kg/m^2 ^and or umbilical circumference > 102 cm; dyslipidaemia (fasting TAG > 1.69 mmol/L and or HDL cholesterol < 1.03 mmol/L) and fasting blood glucose concentration > 6.1 mmol/L. All subjects gave written consent and the study was approved by Curtin University's Human Research Ethics Committee.

Exclusion criteria included smoking and major systemic illness, incorporating gastrointestinal, liver, kidney, diabetes, thyroid, other hormonal diseases and alcoholism. Subjects taking medications that interfere with lipid metabolism and gastrointestinal function were excluded.

Twenty nine subjects participated in the study and were randomised to take either the placebo or fish oil. The fish oil was taken as supplements (Bio-Organics Mega eicosapentaenoic acid (EPA)/docosahexaenoic acid (DHA) capsules, Mayne Group Ltd, Carina, Australia) and the placebo was a 500 mg glucose/starch tablet purpose-made by the School of Pharmacy (Curtin University, Bentley, Australia). Subjects were blinded to the treatment type and were not able to see the alternate treatment during the study (i.e. the difference between the capsules and tablets). Between weeks 0 and 16 subjects consumed four fish oil capsules (providing a total of 1000 mg EPA and 700 mg DHA) or four placebo tablets (2000 mg glucose/starch) per day with meals. Fatty acid composition of the fish oil capsules was confirmed using gas chromatography following esterification with BF_3_-methanol [28]. The initial fish oil intervention period was four weeks since incorporation of fatty acids into membranes occurs within three or four weeks [17,29].

Following the initial four week run-in period with fish oil or placebo, exercise was introduced in combination to both groups. This combined intervention phase was maintained for an additional 12 weeks. The exercise protocol consisted of supervised walking sessions tailored to the subjects' fitness levels. Initially subjects attended a minimum of two sessions per week, which was increased as their fitness increased to 3-5 sessions per week during their final three weeks of intervention. Heart rate (HR) was determined by short range telemetry and moderate intensity exercise was determined as 50 to 65% of the subject's training HR [30]. In each hour-long session subjects expended approximately 1990 kJ of energy. Prior to and at the end of the exercise intervention, subjects underwent a physical work capacity test at 75% of their age predicted maximum HR to determine change to their physical fitness. Apart from the exercise intervention, subjects were asked to maintain their usual physical activity levels throughout the study.

Outcome measures were tested at weeks 0, 4 and 16 where subjects underwent fasting and postprandial assessment. Subjects were instructed to maintain their usual diet throughout the study and this was checked fortnightly by a trained interviewer using food frequency questionnaires. For three days prior to each testing day the diet was standardised. Standard diet formulation was based on the average habitual intake of the subjects determined by three-day food records, as well as data from the Australian National Nutrition Survey [31]. Nutrient composition of the standard diet is shown in Table 1; subjects modified this according to their own energy needs and replicated the same diet before each postprandial test.

**Table 1:** Daily nutrient composition of the standard diet

Energy (kJ)	11 000
Protein (g)	108

Carbohydrate (g)	361

Fat (g)	91

SFA (g)	39

MUFA (g)	35

PUFA (g)	17

Cholesterol (mg)	184

Fibre (g)	42

Table 1 shows the average daily nutrient composition of the diet that subjects were provided with. Subjects modified the amounts of these foods according to their own energy requirements and this diet was replicated before each postprandial test.

### Compliance to intervention

Compliance with the fish oil intervention was assessed by capsule count and subjects who forgot to take their capsules were asked to take them with their following dose. The number of walks completed was similar between groups (37 FOX vs. 40 PX, p = 0.067). Eighty nine percent of the subjects attended > 88% of their walking sessions. On average, subjects walked 38.5 h over 12 weeks and all attended a minimum of three sessions per week in the final three weeks of the intervention. The average percentage HR reserve attained during the exercise was 53.4 ± 1.31% and within the definition of moderate intensity [30]. A 31% increase in physical work capacity was reported followed the exercise intervention (0.95 v. 1.21 W/kg) and this was similar for subjects who received the FO or placebo.

### Postprandial and laboratory assessments

Subjects fasted overnight and at 07.00 hours reported to the clinical room at Curtin University for the postprandial test. Anthropometry was conducted; height was measured using a stadiometer fixed to the wall and weight was determined with the subject wearing light clothing and no shoes. Waist circumference was measured at the level of the umbilicus using a standard metal anthropometry tape.

Venous blood was collected before subjects consumed a moderate-fat test meal (100 g Uncle Toby's Sports Plus™, 150 g skim milk, 30 g skim milk powder and 100 g whipping cream, providing 3688 kJ, 44 g of fat, 94.1 g of CHO and 27.4 g of protein). Fasting blood was analysed for apo B48, lipids (TAG, total-, HDL- and LDL- cholesterol), glucose and insulin. Postprandial samples were collected at 2, 3, 4, 5, 7 and 9 hours in the post-absorptive state and were measured for apo B48 and lipid concentrations. Throughout the day subjects were confined to resting and were given no further food or drink apart from ad libitum water.

Plasma apo B48 was quantified using a Western blotting/enhanced chemiluminescent procedure as previously described [11]. The mean intra- and inter-assay coefficients of variation for apo B48 were each less than 4%.

Plasma TAG and cholesterol concentrations were determined by enzymatic colourimetric kits (Randox Laboratories, Antrim, UK) according to the manufacturers' instructions. Glucose concentration was determined from serum after enzymatic oxidation in the presence of glucose oxidase according to the manufacturer (Randox Laboratories, Antrim, UK). Samples were analysed in triplicate then read photometrically using a Bio-Rad 550 microplate reader.

The insulin assay consisted of a solid-phase, two site chemiluminescent immunometric assay performed on the Biomediq DPC Immulite 2000 analyser (Clinipath Pathology, Perth, Australia). Estimation of insulin resistance was determined by calculation of HOMA score as described by Matthews et al. [32,33].

Baseline data are reported as BP for the placebo group and BF for the fish oil group at week 0. Week four data are reported as P and FO, and week 16 is defined as PX for those on placebo and exercise and FOX for fish oil plus exercise. Summary measures of the postprandial response are reported as the incremental area under the concentration-v.-time curve (_IAUC_). The _IAUC _was calculated by subtracting the area below the baseline concentration from the total _AUC _using the trapezium rule [34].

### Statistical analysis

Within-group changes were compared using paired t-tests and one-way repeated measures ANOVA with Bonferoni's post hoc analysis. The difference between groups was assessed using univariate ANOVA adjusting for age and results at week 0. Statistics were performed using IBM SPSS 19.0 and results were considered significant at a 5% level (two-tailed). Data are presented as mean values with their standard errors.

## Results

Twenty nine subjects were initially recruited. To begin with 16 subjects participated in the fish oil group and after week four, two subjects withdrew for personal reasons. Data for BF and FO are thus reported for 16 subjects and 14 subjects for the FOX intervention. Thirteen subjects completed the entire intervention for the placebo group (BP, P and PX).

Baseline measurements indicate that the subject characteristics were generally similar between the randomised control and intervention groups, although the latter were modestly younger (p = 0.011) (Table 2). The baseline HOMA score suggested insulin resistance, whilst plasma TAG and HDL cholesterol concentrations indicated dyslipidaemia [27].

**Table 2:** Results for baseline, week 4 and week 16

	Control group			Fish oil group		
	**Week 0 (BP)** **(n = 13)**	**Week 4 (P)** **(n = 13)**	**Week 16 (PX)** **(n = 13)**	**Week 0 (BF)** **(n = 16)**	**Week 4 (FO)** **(n = 14)**	**Week 16 (FOX)** **(n = 14)**

Age (y)	56.2 ± 1.3			49.2 ± 2.0^d^		

BMI (kg/m^2^)	31.9 ± 1.3	31.9 ± 1.2	31.6 ± 1.2	32.5 ± 0.8	32.6 ± 0.8	32.3 ± 0.9

UC (cm)	112.8 ± 2.5	111.6 ± 2.2	111.4 ± 2.7	110.1 ± 1.8	108.9 ± 1.7	108.2 ± 1.8

*Fasting results*						

Fasting glucose (mmol/L)	6.49 ± 0.62	6.54 ± 0.73	6.38 ± 0.54	6.50 ± 0.15	6.89 ± 0.88^d^	6.66 ± 0.87

HOMA	3.37 ± 0.42	3.35 ± 0.40	3.09 ± 0.38	3.60 ± 0.34	4.29 ± 0.50^a^	3.27 ± 0.35^c^

Fasting TAG (mmol/L)	1.85 ± 0.24	1.97 ± 0.27	1.85 ± 0.21	1.69 ± 0.15	1.67 ± 0.14	1.31 ± 0.08^b, c, d^

Fasting Total-C (mmol/L)	5.36 ± 0.25	5.60 ± 0.19	5.41 ± 0.29	5.25 ± 0.16	5.40 ± 0.17	5.24 ± 0.20

Fasting LDL-C (mmol/L)	3.55 ± 0.23	3.67 ± 0.16	3.49 ± 0.20	3.39 ± 0.17	3.68 ± 0.17^a^	3.60 ± 0.20

Fasting HDL-C (mmol/L)	0.92 ± 0.05	1.02 ± 0.04^a^	1.06 ± 0.05^b^	1.00 ± 0.03	0.96 ± 0.04	1.04 ± 0.04^c^

Fasting apo B-48 (mg/L)	10.96 ± 1.20	11.12 ± 1.18	10.56 ± 1.11	9.91 ± 0.63	10.17 ± 0.92	8.51 ± 0.82^b^

*Postprandial results*						

TAG _IAUC _(mmol/L/9 h)	9.0 ± 0.8	10.1 ± 1.1	9.0 ± 1.0	9.4 ± 1.0	7.7 ± 1.0^a, d^	6.0 ± 0.8^b, c, d^

Apo B48 _IAUC _(mg/L/9 h)	13.2 ± 2.2	12.5 ± 2.0	14.4 ± 2.0	15.1 ± 2.0	13.4 ± 2.4	14.8 ± 1.9

Results are mean ± SE.

Within group differences as indicated; week 4 v. week 0 ^a^; week 16 v. week 0 ^b^; week 16 v. 4 ^c^. Between group differences placebo v. FO same week ^d^

### Fasting and postprandial lipid and insulin sensitivity results

**Figure 1:**
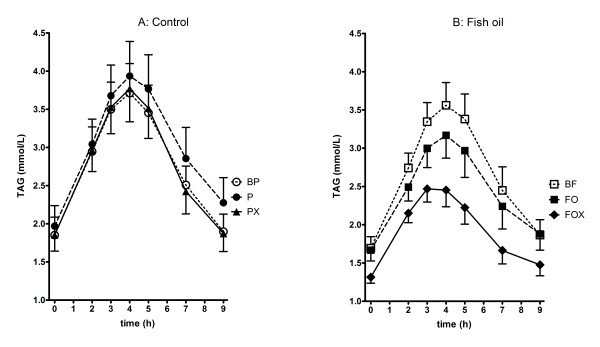
**Postprandial TAG measured at weeks 0, 4 and 16**. BP, placebo group at baseline; P, placebo group at 4 weeks; PX, placebo plus 12 weeks of exercise. BF, fish oil group at baseline; FO, four weeks of fish oil; FOX fish oil plus 12 weeks of exercise.

Fish oil supplementation provided at 1000 mg EPA and 700 mg DHA per day for four weeks in insulin resistant men did not lower fasting plasma TAG, however postprandial lipaemia expressed as TAG_IAUC _was reduced by almost 20% compared to baseline (p = 0.020) and 25% less than subjects given placebo capsules (p = 0.025) (Table 2, Figure 1). There was no difference in fasting TAG concentration or TAG_IAUC _in subjects given placebo. The improvement in TAG_IAUC _in FO supplemented subjects occurred despite a higher blood glucose (p = 0.034) and HOMA score (p = 0.034). Total fasting cholesterol remained unchanged in the FO group, however LDL-cholesterol concentration was increased by approximately 8% compared to baseline (p = 0.049) (Table 2). There was no significant difference in total- or LDL-cholesterol concentrations between subjects given FO or placebo.

Introduction of exercise concomitant with FO supplementation had a substantial lowering effect on fasting plasma TAG concentration (p = 0.026) and TAG_IAUC _(p = 0.004) that was not apparent with the provision of exercise without FO supplementation. In the FOX group of subjects, fasting TAG concentration was reduced by 21% after exercise (p = 0.026), whereas TAG_IAUC _was reduced 36% compared to baseline (p = 0.025) and by 22% compared to FO supplementation alone (p = 0.004) (Table 2). Exercise did not reduce total- or LDL-cholesterol concentrations but did increase HDL-cholesterol concentration to a similar extent in both groups of subjects (PX vs. P, p = 0.025; FOX vs. FO, p = 0.001). Moreover, the addition of exercise completely abolished the heightened HOMA score induced by FO supplementation alone (p = 0.020).

#### Fasting apo B48 and postprandial apo B48

**Figure 2:**
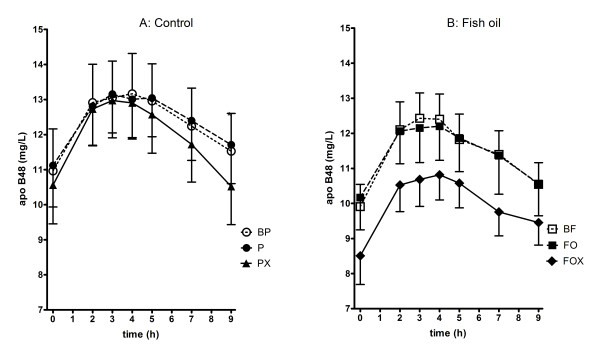
**Postprandial apo B48 measured at weeks 0, 4 and 16**. BP, placebo group at baseline; P, placebo group at 4 weeks; PX, placebo plus 12 weeks of exercise. BF, fish oil group at baseline; FO, four weeks of fish oil; FOX fish oil plus 12 weeks of exercise.

Apo B48 is an unequivocal marker of chylomicron metabolism and was used to differentiate between the putative lipid modulating effects of FO and exercise versus that of chylomicron kinetics. Of the interventions investigated, only the combination of FOX attenuated the fasting apo B48 concentration (p = 0.049). There was no significant effect of FO, PX or FOX on the postprandial chylomicron apo B48_IAUC _response (Figure 2, Table 2).

## Discussion

This paper reports on the effects of fish oil and chronic exercise on chylomicron and lipid metabolism in men with insulin resistance. Subjects taking fish oil showed a reduced postprandial TAG response to the moderate fat-containing meal after four weeks. Following the 12 week walking program the fish oil group exhibited an even greater postprandial TAG reduction, and a lower fasting TAG concentration. The combined interventions did not decrease the total or LDL cholesterol concentrations, however by the end of the treatment both groups had increased HDL-cholesterol concentrations compared to baseline and each individual treatment at week four. Most significantly our findings suggest that the combined effect of the chronic exercise and fish oil was sufficient to alter the basal metabolism of chylomicrons in these viscerally obese subjects.

The reduction of fasting TAG that occurred when the interventions were combined corresponds with suggestions in the literature that both exercise and fish oil could individually reduce VLDL production [15,35], mainly by suppressing hepatic lipogenesis. The combined interventions also lowered the postprandial TAG response. A reduction in fasting VLDL production could have facilitated the lower postprandial TAG_IAUC _response as a result of less competition with the exogenous TAG for lipid clearance mechanisms. The lipaemic reduction is also likely because of increased lipoprotein lipase activity in adipose tissue following fish oil [36] or in the leg muscle after exercise [24]. As a result of chronic training adaption, exercise is also suggested to promote lipid clearance by increasing capillary vascularisation [37,38], as was expected after 12 weeks of regular walking. Since chylomicrons are the preferred substrate for lipoprotein lipase, it is likely that our combined interventions affected chylomicron delipidation in the early postprandial phase and then the VLDL that predominates later in the postprandial excursion [5]. Hydrolysis from both hepatic and intestinal lipoproteins is likely to be a pivotal mechanism in the postprandial TAG reduction.

The effect of fish oil significantly lowered the TAG_IAUC _response even without exercise. In animals and humans, fish oil accelerates catabolism of VLDL to LDL [36] through enhancing blood circulation to capillary lipases, lipase binding or expression [39-41]. Such catabolic changes correspond to our results of an increased LDL cholesterol concentration initially after four weeks of fish oil. Others reporting similar findings after four and six weeks of fish oil have proposed that this increase might be in the LDL_1 _fraction rather the small dense particles [42,43] which suggests that measuring plasma lipids following fish oil in the longer term (i.e. chronic interventions) may be more physiological useful than shorter studies. Interestingly we also reported increases in glucose concentration and HOMA score with the four weeks of fish oil, findings which are also reported in the literature. Woodman et al. [44] measured interim blood glucose concentrations at three weeks during their six week n-3 FA trial and discuss a transient adverse trend in glycaemic control, which was corrected by the end of the trial. As did Mori and colleagues [45], who suggest that the variability in the effect of fish oil on glycaemic control may arise from varying degrees of insulin sensitivity between subjects and the presence of other disorders including hypertension and obesity. By the time our subjects had undergone the additional exercise at week 16, the fish oil-associated HOMA increase was abolished, consistent with earlier findings [46]. However based on the present results, it would be prudent to suggest that insulin resistant patients monitor their glycaemic control when commencing fish oil supplements, and incorporate regular, moderate intensity exercise into their lifestyle.

Our key finding was that fish oil plus chronic exercise did modulate the basal metabolism of apo B48, a finding not reported elsewhere in the literature. This finding is important since chylomicron homeostasis is more difficult to modulate than that of hepatic lipoproteins [25]. The fasting apo B48 concentration represents the difference between the constitutive secretion rate and the clearance rate of the lipid-poor remnants. Suggestions from cell, animal and human studies is that fish oil and exercise could modulate both the clearance of apo B48 remnants [22,47-52], and also play a role in reducing apo B48 secretion [53].

Our results have shown that a combined fish oil and exercise intervention may be effective in reducing fasting apo B48 in otherwise free-living, insulin resistant subjects. The interaction between fish oil and exercise may be hinged on the fact that our subjects were insulin resistant throughout the study. Insulin resistance may have contributed to an over-secretion of intestinal apo B [5] as well as compromised the receptor-mediated uptake of apo B48 in our subjects. Another feature of insulin resistance is the association with inflammatory markers (e.g. C-reactive protein, prostaglandin PGE_2_, pro-inflammatory cytokines) [54] and high oxidative stress [55]. Liver cell culture studies suggest that this stress may impact chylomicron remnant uptake [52]. Therefore it is plausible that by improving our subjects' oxidative status through 12 weeks of regular exercise [56,57], and long-term fish oil [58-61] we may have somewhat corrected the defective uptake of chylomicron remnants by the liver in separate but related mechanisms. A further suggestion from cell culture studies is that when the oxidative status of the liver was improved, the degree of improvement to remnant uptake was even more enhanced when remnants were enriched with n-3 FA [52]. This suggestion might allude to why in our human study the basal apo B48 concentration was only lower when the fish oil and chronic exercise were added together.

We therefore conclude that an effective therapeutic strategy for the chylomicronaemia in subjects with MetS is the combination of chronic, regular exercise and a relatively low dose of fish oil. We also note that these benefits occurred even without insulin resistance being completely normalised. The mechanisms through which these two interventions work are likely to be different, which may be why they were efficacious only when used in combination. While our study was not intended to test the mechanisms, further investigation is warranted to decipher the reasons that chronic exercise and fish oil reduce basal apo B48 concentration in insulin resistant men.

## Abbreviations

Apo: apolipoprotein; AUC: area under the curve; BF: fish oil group at baseline; BMI: body mass index; BP: placebo group at baseline; CHO: carbohydrate; DHA: docosahexaenoic acid; EPA: eicosapentaenoic acid; FA: fatty acid; FO: fish oil; FOX: fish oil + exercise; HDL: high density lipoprotein; HOMA: homeostatic model measurement; HR: heart rate; IAUC: incremental area under the curve; LDL: low density lipoprotein; MetS: metabolic syndrome; P: placebo; PX: placebo + exercise; TAG: triacylglycerol; VLDL: very low density lipoprotein

## Competing interests

The authors declare that they have no competing interests.

## Authors' contributions

KS was responsible for conducting the study and sample assays, data analysis and drafting the manuscript. JM conceived the study and all authors participated in its design. JM and TJ critically revised the manuscript. All authors read and approved the final manuscript.
